# The who, what, where, how, and why of endoscopic submucosal dissection in Canada: A survey among Canadian endoscopists

**DOI:** 10.1002/jgh3.12526

**Published:** 2021-03-08

**Authors:** Mandip Rai, Douglas Motomura, Lawrence Hookey, Mainor R Antillon, Robert Bechara

**Affiliations:** ^1^ Department of Medicine, Division of Gastroenterology Queen's University Kingston Ontario Canada; ^2^ Department of Medicine, Division of Gastroenterology Mayo Clinic Health System Eau Claire Wisconsin USA

**Keywords:** Canada, endoscopy, endoscopic submucosal dissection

## Abstract

**Background and Aim:**

Endoscopic submucosal dissection (ESD) is an internationally accepted technique for the resection of superficial gastrointestinal neoplasia. ESD allows for en‐bloc removal when endoscopic mucosal resection (EMR) is unsuitable due to the size or depth of the lesion. The aim of this survey was to examine Canadian clinicians' experience and perceptions of ESD as its prevalence increases across the country.

**Methods:**

An electronic survey consisting of 24 multiple‐choice questions was distributed via the Canadian Association of Gastroenterology email database and directly to those known to be performing or interested in ESD. The survey covered training, practice, obstacles in implementation, and perceptions of the future of ESD in Canada.

**Results:**

A total of 21 participants completed the survey. ESD was performed primarily in the endoscopy suite exclusively (71%), and most operators (64%) performed it on an outpatient basis. Procedure time was selected as the greatest technical challenge in the performance of ESD by 86% of the participants. Both lack of formalized training and long procedure times were the highest ranked barriers to the adoption of ESD. Over the next 5 years, 95% believed there would be an increase in ESD volume in Canada, and 43% believed ESD was ready for adoption by more therapeutic endoscopists.

**Interpretation:**

In this survey, we explored the current practice, attitude, and challenges of ESD in the Canadian landscape. As the performance of ESD increases and gains more acceptance across Canada, there are opportunities to address technical challenges and barriers through the formalization of training, education, and practice guidelines.

## Graphical abstract

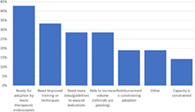

## Introduction

Endoscopic submucosal dissection (ESD) was developed in Japan in the 1990s as a novel technique that allowed en‐bloc removal of early gastric neoplasia regardless of size. Subsequently, its indications expanded to superficial neoplasia in the esophagus and colon.^1^ ESD allows for en‐bloc removal of neoplasia when en‐bloc EMR is not possible due to the size or invasion depth of the lesion. In the last 30 years, the technique has been refined in Eastern countries and is now widely used. Currently, Japanese, American, and European societies provide guidelines for the indications of ESD; however, no such Canadian guidelines exists.^1^, ^2^, ^3^

The current practice, attitude, and challenges of ESD in Canada are largely unknown as there is no current literature examining this. The aim of this study was to gain a fundamental understanding of the scope of ESD practice in Canada, with a focus on operator demographics, current practice, challenges, and predicted growth. The knowledge gained could thereby aid in establishing a framework to ensure safe and effective use and growth of ESD within Canada.

## Methods

### Survey design

A list of 24 questions was prepared by a therapeutic endoscopist with 5 years of experience performing ESD independently in Canada (R B). The survey covered the operator demographics, training, practice, challenges in performance/implementation, and the future of ESD in Canada. Survey items were presented as multiple‐choice nominal responses. Participants had the option of free‐text entry responses if a satisfactory option was not presented.

### Recruitment

The survey was intended for recipients who perform or were interested in performing ESD. An electronic anonymous survey consisting of multiple‐choice questions regarding ESD in Canada was disseminated via the Canadian Association of Gastroenterology (CAG) and directly to endoscopists known to be performing or interested in ESD. The survey was sent to the CAG members through their email database, which is roughly approximately 11 000 members. The survey was included in an email from the CAG, where multiple other surveys were also presented. Members perused and chose to participate in surveys of interest. The link to the anonymous survey was also sent directly to 11 Canadian endoscopists known to be performing ESD or interested in ESD. Those interested were known from an ESD special interest group that was created in CAG. SurveyMonkey (SurveyMonkey, San Mateo, California, USA) was used as the platform of distribution. Data were collected from May 2018 to November 2018, and renumeration was not provided for survey completion.

### Analysis

Responses were collected and aggregated in Microsoft Excel. The data were analyzed and presented qualitatively.

## Results

### Respondent demographics

Twenty‐one endoscopists from various institutions completed the survey in addition to 20 gastroenterologists and one thoracic surgeon. Nine are practicing in Ontario, four in Quebec, four in Alberta, two in British Columbia, one in Saskatchewan, and one in Newfoundland.

### Baseline knowledge and ESD training

Of the 21 respondents, 13 (62%) received ESD training after completion of core education. Of the 13, 12 disclosed the duration of ESD training, and 9 disclosed the number of ESDs performed during training. The training methods included American Society of Gastrointestinal (ASGE)‐accredited courses; overseas fellowships; North American fellowships; and a mixture of learning, which also included animal labs and receiving mentorship. During training, a median of 11 (range 0–55) ESD procedures was performed, with the majority occurring in the stomach (median 5, range 0–10) or rectum (median 1, range 0–10) (Table 1).

**Table 1:** Endoscopic submucosal dissection (ESD) training

ESD training (*n* = 21)	
Fellowship in North America	2 (10%)
Fellowship Overseas	4 (19%)
ASGE or other accredited courses	6 (29%)
Other†	1 (5%)
Did not pursue further formal ESD training	8 (38%)
Duration of ESD training (*n* = 12)	
Less than 3 months	5 (42%)
Between 3 and 6 months	3 (25%)
Between 6 and 12 months	2 (17%)
Between 12 and 24 months	1 (8%)
Greater than 24 months	1 (8%)
Location and number of ESDs performed during training (*n* = 9, median, range)	
Esophagus	1 (0–40)
Stomach	5 (0–10)
Colon	0 (0–5)
Rectum	1 (0–10)
Duodenum	0 (0–0)
Total	11 (0–55)

^†^ Respondent noted training included “ASGE course, animal labs, spent time in Japan, [and] had Japanese mentor come to Canada”.

### Current state of ESD practice

**Figure 1:**
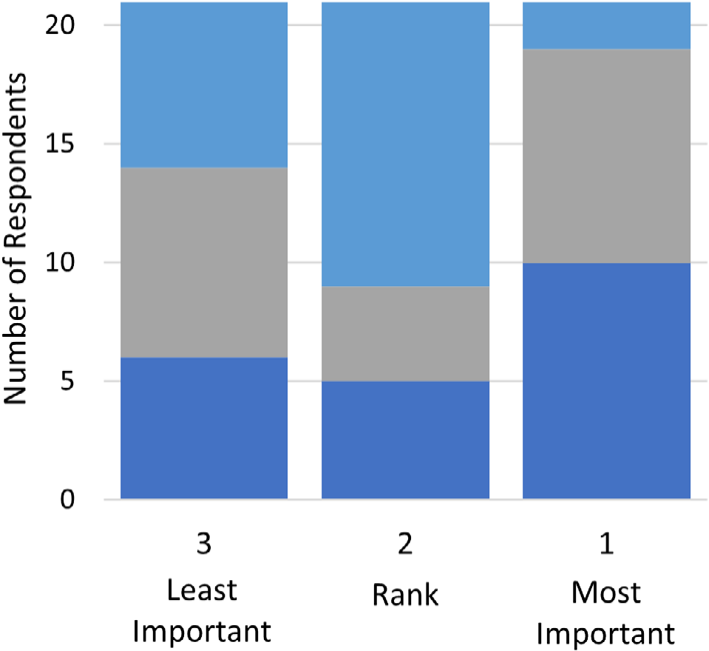
Rationale for endoscopic submucosal dissection performance ranking. 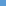, lower recurrence rates; 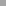, avoiding surgery; 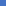, en‐bloc resection.

**Figure 2:**
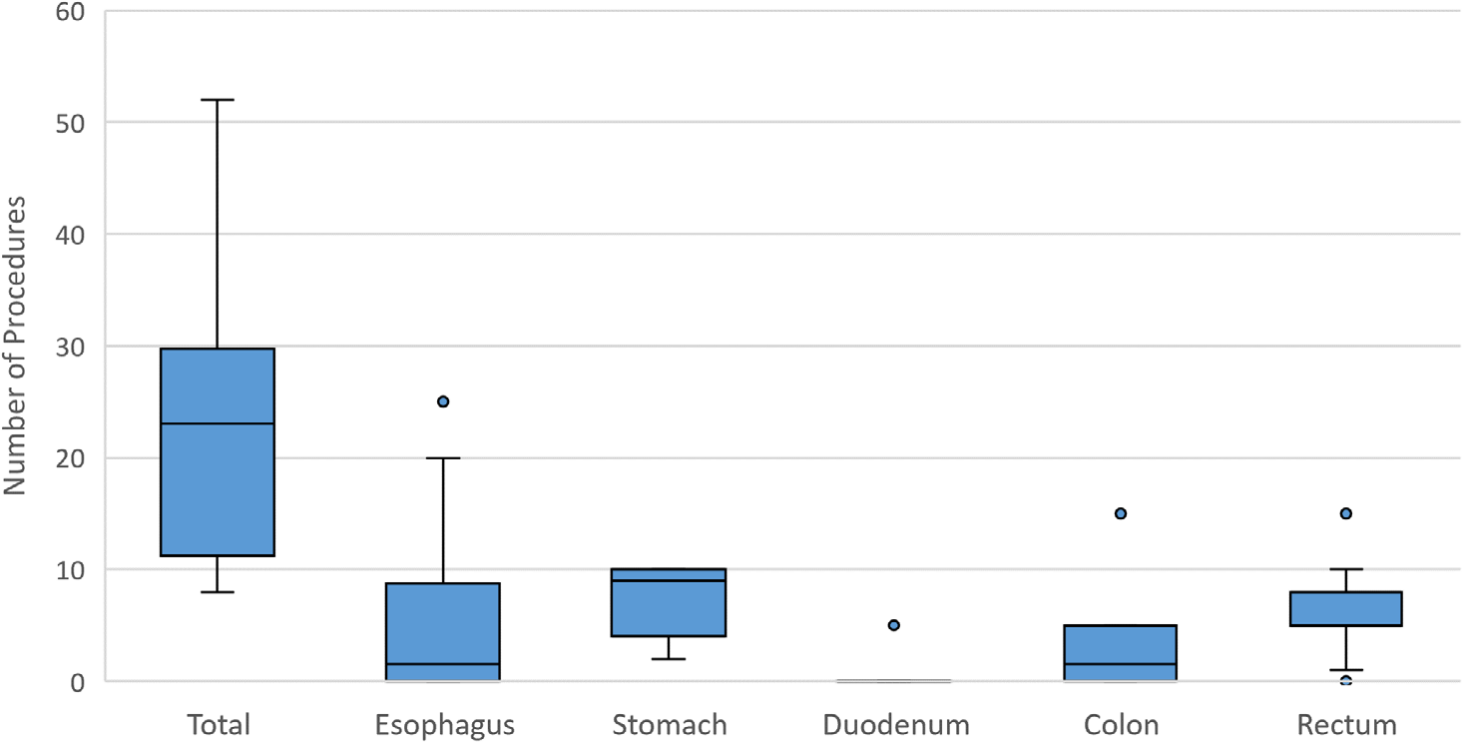
The estimate number of ESDs performed annually by ESD operators (*n* = 9). Boxes represent median and Interquartile Range (IQR), whiskers present the range. ESD, endoscopic submucosal dissection.

In Table 2, the current state of ESD practice with respect to operator demographics, rationale for performance, procedure location, and techniques employed are reviewed. Most respondents (80%) noted that gastroenterologists were exclusively performing ESD at their centers. ESD is performed primarily in the endoscopy suite exclusively (71%), and most operators (64%) performed it on an outpatient basis. The highest ranked reasons for performance of ESD were avoidance of surgery and the requirement for en‐bloc resection (Fig. 1). Eleven (85%) respondents were noted to be performing ESD, with six (55%) in Ontario, three (27%) in Quebec, and two (18%) in British Columbia. Ten (91%) of the operators were gastroenterologists, and one (9%) was a thoracic surgeon. Nine operators disclosed their annual estimated ESD volume with a median of 21 cases, most of which were gastric (median 8, range 2–10) or rectal (median 5, range 0–15) (Fig. 2). The majority of ESD operators (91%) noted performing hybrid ESD (usage of a snare after complete marginal incision and partial submucosal dissection) less than 10% of the time, with the remainder only performing it between 10 and 50% of the time.

**Table 2:** Current state of endoscopic submucosal dissection (ESD) practice

Specialty performing ESD (*n* = 15)	
Solely by gastroenterology	12 (80%)
Solely by surgery	1 (7%)
Both gastroenterology and surgery	2 (13%)
Location where ESD is performed (*n* = 14)	
Endoscopy suite exclusively	10 (71%)
Operating room exclusively	3 (21%)
Both the endoscopy suite and operating room	1 (7%)
Frequency of using tissue retraction techniques (*n* = 10)	
Always	0 (0%)
Usually	0 (0%)
Sometimes	4 (40%)
Rarely	3 (30%)
Never	3 (30%)
Injection solution used (*n* = 11)	
Hetastarch (i.e. Voluven, Hespan)	8 (73%)
Glycerol	1 (9%)
Other^†^	2 (18%)
Degree of satisfaction with injection solution (*n* = 11)	
Very satisfied	7 (64%)
Somewhat satisfied	4 (36%)
Not satisfied	0 (0%)

^†^ Respondents noted use of hydroxypropyl methycellulose or hylauronic acid in the form of eyedrops.

The primary injection solution used during ESD was reported to be Hetastarch (i.e. voluven, Hespan) (73%) with glycerol, hydroxypropyl methylcellulose, and hyaluronic acid noted as other choices. Most operators (64%) were very satisfied with their injection solution. None of the operators routinely implemented tissue retraction techniques.

The operators estimated performing a median of 21 cases annually, most of which were gastric (median 8, range 2–10) or rectal (median 5, range 0–15) (Fig. 1).

### Current perceptions of ESD and its future in Canada

**Figure 3:**
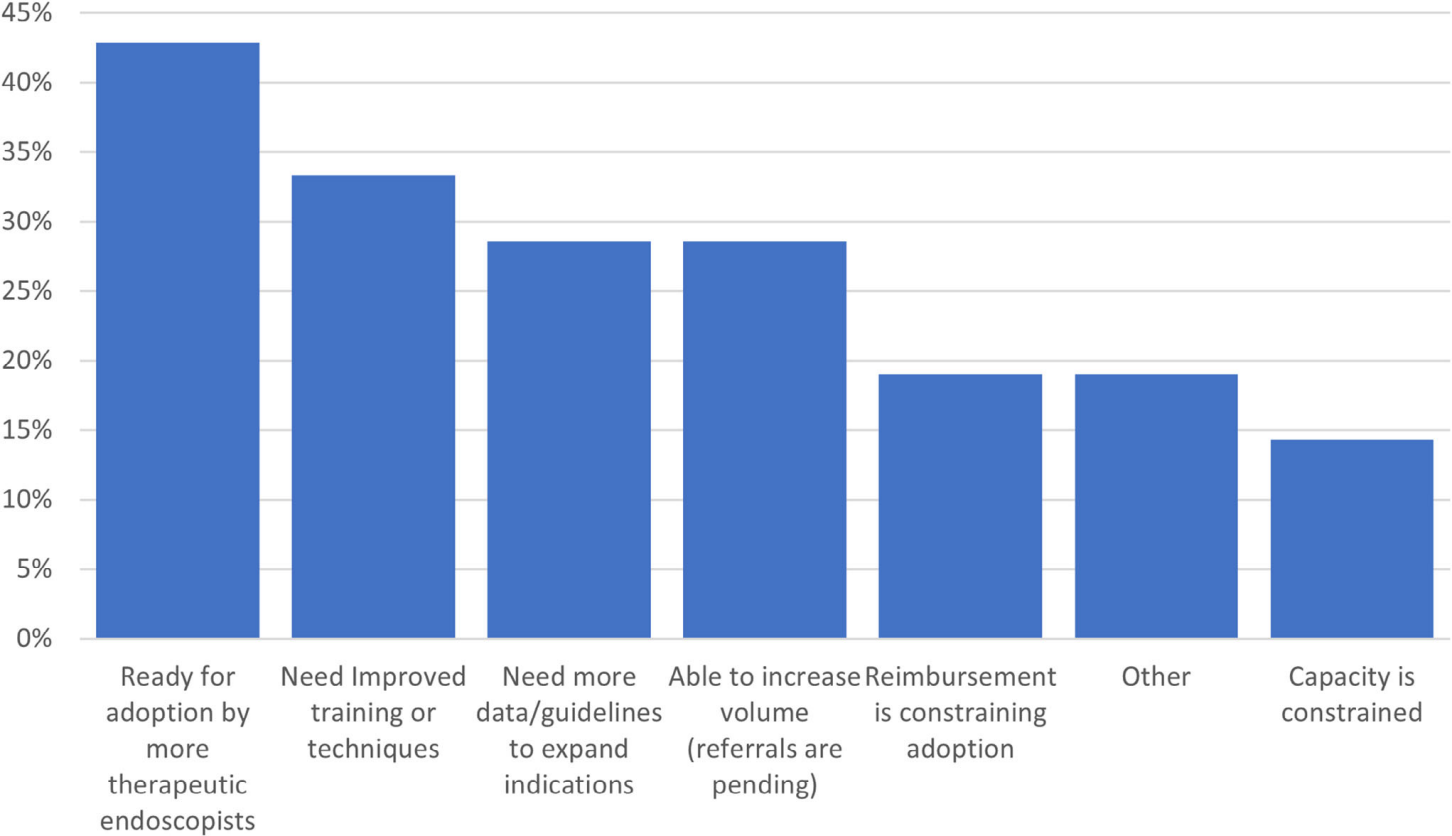
Perception of readiness to adopt endoscopic submucosal dissection in Canada.

The following results illustrate the perceptions of Canada's readiness to adopt ESD, barriers to its performance, and its role in practice in the coming years. With respect to the adoption of ESD (multiple answers permitted), the most selected response was that “ESD is ready for adoption by more therapeutic endoscopists” (43%), followed by “[there is a] need for improved training/techniques before significant increase in adoption (33%)” (Fig. 3).

**Figure 4:**
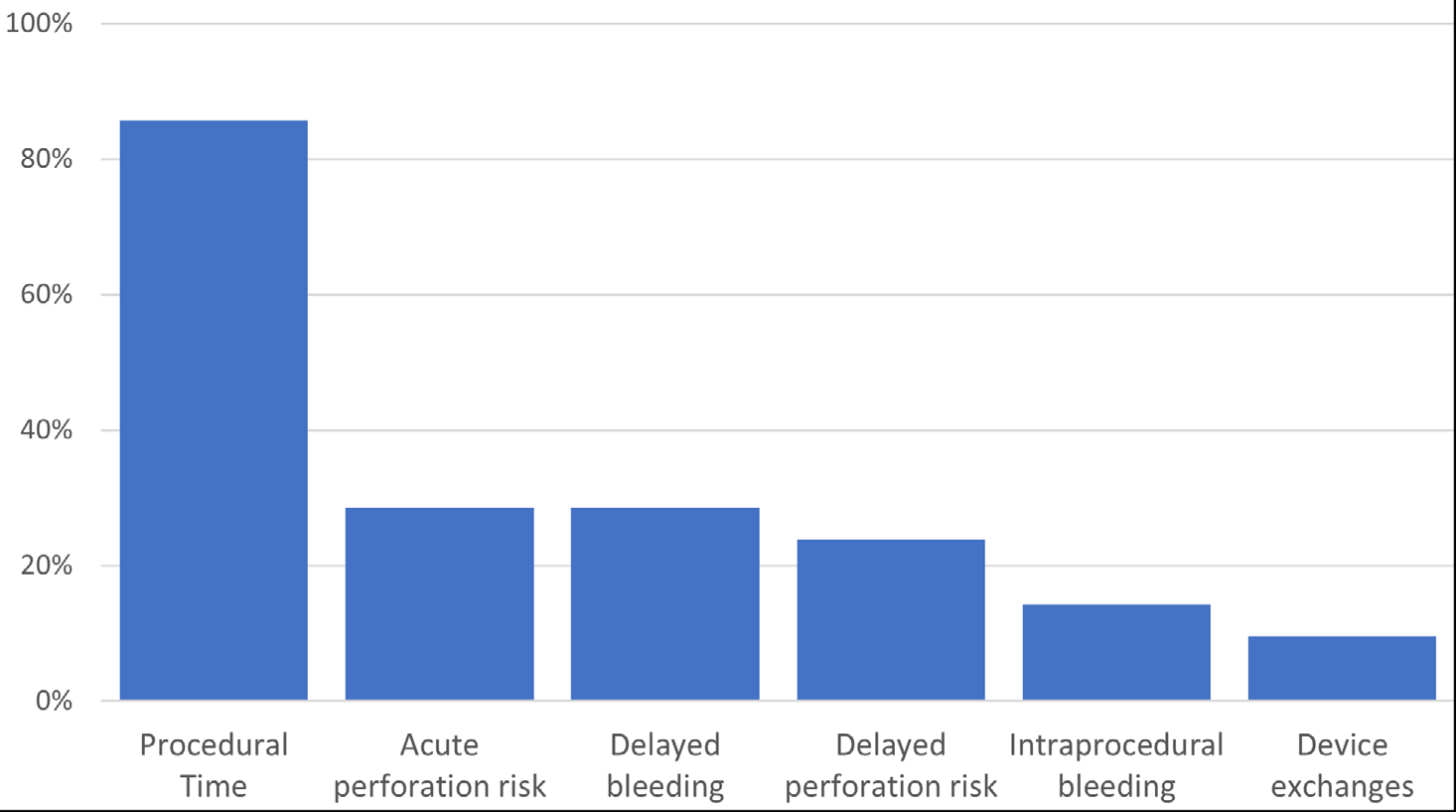
Technical challenges to performing endoscopic submucosal dissection today (multiple answers permitted) *n* = 21.

Length of procedure time was the most selected (86%) technical challenge of ESD, followed by the risk of acute perforation (29%) and delayed bleeding (29%) (Fig. 4). Twenty (90%) of the respondents believed there would be an increase in ESD volumes within the next 5 years, 10 (48%) of whom believed it would be a dramatic increase.

**Figure 5:**
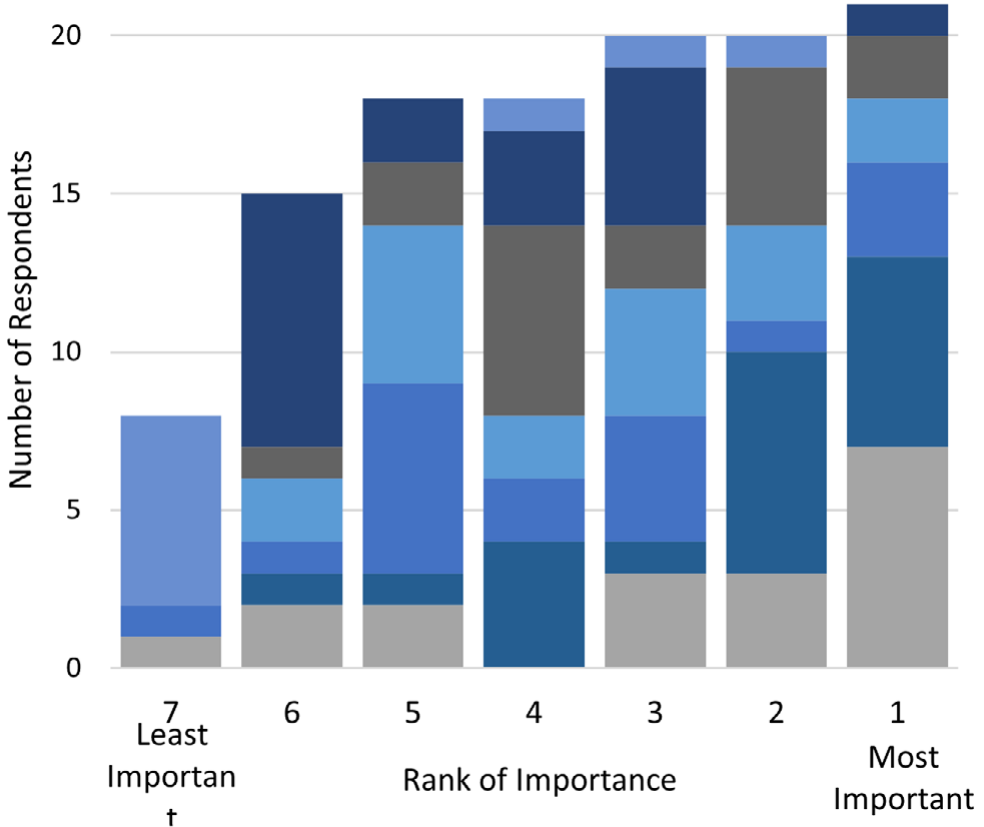
Ranking of perceived barriers to adopting endoscopic submucosal dissection in Canada. 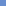, Other; 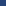, lack of society guidelines; 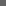, low procedure volume; 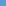, lack of endoscopic diagnosis; 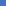, lack of reimbursement; 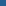, long procedural times; 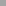, lack of training.

**Figure 6:**
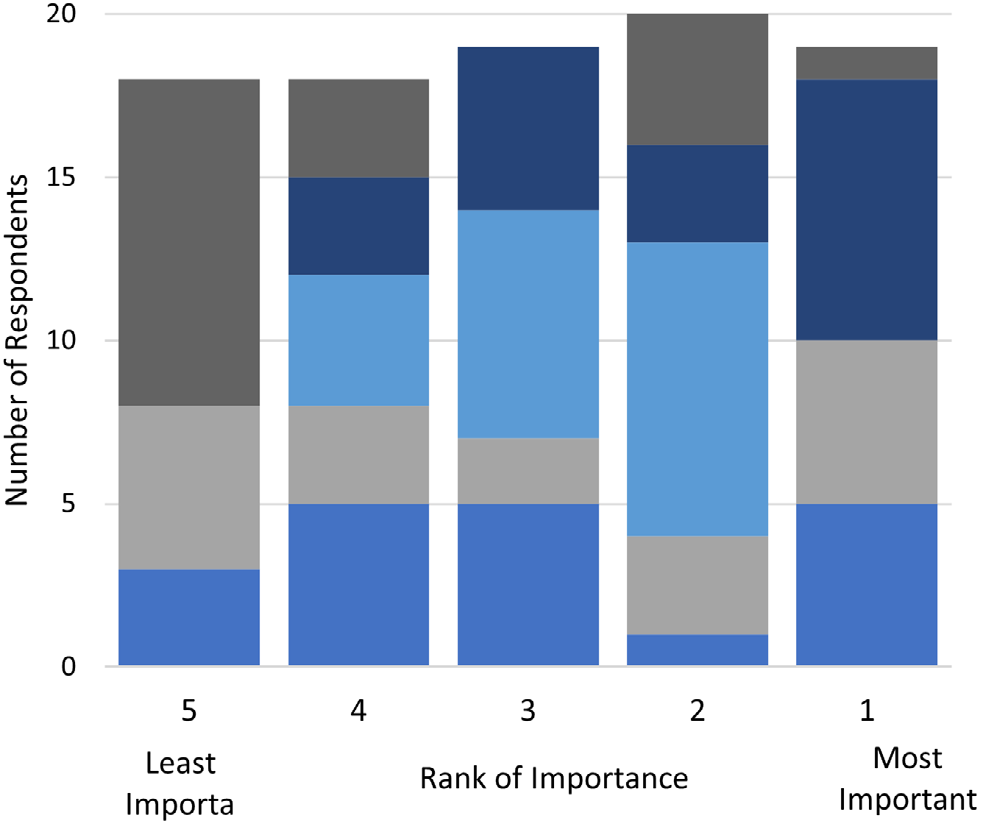
Ranking of the proposed measures for facilitating endoscopic submucosal dissection (ESD) adoption in Canada. 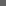, Creation of ESD registry; 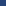, more access to ESD experts; 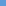, non‐CAG ESD courses; 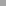, CAG ESD courses; 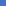, better accessories. CAG, Canadian Association of Gastroenterology.

Regarding current barriers to ESD uptake, lack of training availability was the highest ranked (33%), followed by long procedure times (29%) and lack of reimbursement (14%) (Fig. 5). To improve the adoption of ESD, the highest ranked solution was to improve access to ESD experts (42%) followed by the desire for a CAG‐endorsed ESD course (26%) and access to better tools/accessories (26%) (Fig. 6).

## Discussion

In this survey, we gained a preliminary understanding of the current practice and attitude toward ESD in the Canadian landscape. As the uptake of ESD was in its early stages at the time of the survey, of the 21 respondents, 11 reported actively performing ESD. One participant identified as being able to perform ESD but was not yet performing it at his or her center as the operators were still receiving training. We speculate that there has been a significant increase in the number of ESD operators since the survey was completed.

Most survey respondents were from Ontario and Quebec, Alberta, or British Columbia (91%), and this corresponds to the general population distribution in Canada, with Ontario being the most populous province, followed by Quebec, British Columbia, and Alberta.

Despite most operators pursuing additional training for ESD, the cumulative training time was less than 3 months for 42% and 6 months or less for 67%. Similar to the minimum standards for other advanced procedures, there have been publications on the minimal standards that should be met to achieve ESD competence. One proposed pathway involves beginning on animal models, proceeding to an intensive observation period, and progression to simple cases in the rectum and stomach.^4^ The European Society of Gastrointestinal Endoscopy (ESGE) has made recommendations, with at least 20 procedures in animal models with the goal of 80% R0 resection rate and 0% perforations in 10 consecutive cases before proceeding to training in patients. When this target has been achieved in the animal model, the trainee will observe 20 procedures in a tertiary care center before assisting with 5 ESD procedures. Subsequently, the next 10 human ESDs performed (per organ) would be directly supervised by an ESD‐proficient endoscopist.^5^ To promote the continued growth of ESD in Canada while ensuring procedural safety and quality Canadian guidelines would be a valuable resource.

The American Gastroenterological Association (AGA) has made recommendations for the appropriate indications for ESD.^3^ The indications vary slightly based on anatomic site but generally include lesions with severe fibrosis and/or high‐grade dysplasia to superficial submucosal cancer that cannot be removed en bloc with EMR. Most respondents were performing ESD on primarily intramucosal cancer. Despite no published Canadian guidelines, it appears the reported performance of ESD in Canada falls within recommended international guidelines.

On assessment of potential barriers to the adoption of ESD in Canada, procedural time was at the forefront. Of the respondents, 29% ranked procedural time as the largest barrier to adoption, with an average rank of 2.5 of 7. Lack of a formalized training program (discussed above) was ranked by 33% of respondents as the largest barrier to adoption, with an average rank of 2.8. Operative time of ESD will remain longer than most endoscopic procedures given the inherent nature of ESD, but a decrease in procedural time is expected as experience grows. Total operative time is used as a measure of procedural competency and has been shown to improve within the first 30 cases.^6^ A recent Canadian abstract demonstrated a 134% improvement in procedural velocity from the operator's first 10 ESDs to their last 10 cases (from 75 to 32 min/cm).^7^ Decreased operative time is also possible with simple technical modifications, such as tissue retraction. A wide variety of retraction techniques exist, ranging from the simple usage of clips and sutures to dual endoscopic technique.^8^, ^9^ These techniques allow for better delineation of tissue planes and maintenance of field of view. Furthermore, significant decreases of procedure time have been demonstrated.^10^, ^11^ None of the respondents reported regular use of tissue retraction techniques. This demonstrates the necessity for education of ESD techniques in Canada as some of the techniques are easy and cost‐effective to implement using standard equipment that can readily decrease procedure time.^10^

Low procedural volumes continue to be an issue regarding maintenance of competency. Of the nine respondents who disclosed estimated annual volumes, the median number of procedures performed was 21 (IQR 11–29). A systematic review and meta‐analysis revealed that low volume centers (defined as <24 ESDs performed per year, or <2 per month) had a significantly higher rate of serious adverse events compared to high volume centers (1.9 *vs* 0.7%).^12^ This has led to the ESGE recommending at least 25 procedures per year to maintain competency.^5^ To support this, the performance to ESD would likely be limited to centers of excellence to support the minimum volume to maintain competence. Despite these perceived barriers, most participants expect an increase in ESD volumes in the next 5 years.

This study has some limitations. Surveys are inherently susceptible to bias and inflexibility. The survey was sent out through the CAG email database with approximately 11 000 members. As the target population was those performing or interested in ESD, it was impossible to estimate our response rate. Thus, we suspect that the overall low response rate likely reflects the early stages of implementation and dissemination of ESD in Canada. Moreover, those who chose to respond to this nonmandatory survey likely have vested interest and optimism in the growth of ESD. However, this likely represents a population of Canadian gastroenterologists and surgeons who are most familiar and acquainted with the benefits and barriers of the procedure and is, thus, a likely accurate view of the current climate and future of ESD in Canada.

The initial dominant usage of ESD in Canada will differ from that in Asia. With less gastric and esophageal squamous neoplasia, it is likely that Canadian training, and eventual practice, will be focused on more colorectal and Barrett's associated neoplasia. This has been shown to be a viable training method in the absence of early gastric cancers.^13^ Concurrently, uptake of advanced endoscopic diagnostic techniques, including image‐enhanced endoscopy, will be imperative to ensure the selection of appropriate lesions for ESD.^1^ Finally, knowledge dissemination to endoscopists, surgeons, specialized gastrointestinal pathologists, and oncologists is paramount to ensure ESD continues to expand in Canada and is utilized in the most appropriate candidates.

## Supporting information


**Appendix S1**. ESD in Canada Survey Questions.

## References

[1] Pimentel‐Nunes P , Dinis‐Ribeiro M , Ponchon T *et al*. Endoscopic submucosal dissection: European Society of Gastrointestinal Endoscopy (ESGE) Guideline. Endoscopy. 2015; 47: 829–54. 26317585 10.1055/s-0034-1392882

[2] Ono H , Yao K , Fujishiro M *et al*. Guidelines for endoscopic submucosal dissection and endoscopic mucosal resection for early gastric cancer. Dig. Endosc. 2016; 28: 3–15. 26234303 10.1111/den.12518

[3] Draganov PV , Wang AY , Othman MO , Fukami N . AGA Institute Clinical Practice update: Endoscopic Submucosal Dissection in the United States. Clin Gastroenterol Hepatol. 2019; 17: 16–25.e1. 30077787 10.1016/j.cgh.2018.07.041

[4] Draganov PV , Coman RM , Gotoda T . Training for complex endoscopic procedures: how to incorporate endoscopic submucosal dissection skills in the West? Expert Rev. Gastroenterol. Hepatol. 2014; 8: 119–21. 24308749 10.1586/17474124.2014.864552

[5] Pimentel‐Nunes P , Pioche M , Albéniz E *et al*. Curriculum for endoscopic submucosal dissection training in Europe: European Society of Gastrointestinal Endoscopy (ESGE) position statement. Endoscopy. 2019; 51: 980–92. 31470448 10.1055/a-0996-0912

[6] Oda I , Odagaki T , Suzuki H , Nonaka S , Yoshinaga S . Learning curve for endoscopic submucosal dissection of early gastric cancer based on trainee experience. Dig Endosc. 2012; 24(Suppl 1): 129–32. 22533768 10.1111/j.1443-1661.2012.01265.x

[7] Trasolini R , Zhao B , Chahal D , Lam E . A89 implementing endoscopic submucosal dissection in a western Canadian setting: outcomes, learning curve and logistical considerations. J Can Assoc Gastroenterol. 2020; 3(Supplement_1): 103–4.32395684

[8] Imaeda H , Hosoe N , Kashiwagi K *et al*. Advanced endoscopic submucosal dissection with traction. World J Gastrointest Endosc. 2014; 6: 286–95. 25031787 10.4253/wjge.v6.i7.286 PMC4094986

[9] Oyama T . Counter traction makes endoscopic submucosal dissection easier. Clin Endosc. 2012; 45: 375–8. 23251884 10.5946/ce.2012.45.4.375 PMC3521938

[10] Yoshida M , Takizawa K , Nonaka S *et al*. Conventional versus traction‐assisted endoscopic submucosal dissection for large esophageal cancers: a multicenter, randomized controlled trial (with video). Gastrointest Endosc. 2020; 91: 55–65.e2. 31445039 10.1016/j.gie.2019.08.014

[11] Suzuki S , Gotoda T , Kobayashi Y *et al*. Usefulness of a traction method using dental floss and a hemoclip for gastric endoscopic submucosal dissection: a propensity score matching analysis (with videos). Gastrointest. Endosc. 2016; 83: 337–46. 26320698 10.1016/j.gie.2015.07.014

[12] Fuccio L , Hassan C , Ponchon T *et al*. Clinical outcomes after endoscopic submucosal dissection for colorectal neoplasia: a systematic review and meta‐analysis. Gastrointest Endosc. 2017; 86: 74–86.e17. 28254526 10.1016/j.gie.2017.02.024

[13] Yang D‐H , Jeong GH , Song Y *et al*. The feasibility of performing colorectal endoscopic submucosal dissection without previous experience in performing gastric endoscopic submucosal dissection. Dig. Dis. Sci. 2015; 60: 3431–41. 26088371 10.1007/s10620-015-3755-0

