# Supplementary material for: The who, what, where, how, and why of endoscopic submucosal dissection in Canada: A survey among Canadian endoscopists
Source: JGH Open. 2021 Mar 8;5(7):734–9. doi: 10.1002/jgh3.12526 (PMC8264244; doi:10.1002/jgh3.12526)
Supplement: Supplementary file 1 — Appendix S1. ESD in Canada Survey Questions. [file JGH3-5-734-s001.docx]

**ESD in Canada Survey Questions**

**Q1** Select your location

- (province)

**Q2** Estimated number of ESD operators in your in your province

(free text)

**Q3** Estimated ESD procedures (annually) in your hospital

- (free text)

**Q4** How would you describe yourself?

- Endoscopist-Gastroenterology trained
- Endoscopist-Thoracic Surgery trained
- Endoscopist-Upper GI Surgery trained
- Endoscopist-Hepatobiliary Surgery trained
- Endoscopist-Colorectal Surgery trained
- Other (please specify)

**Q5** Where are your ESD procedures performed?

- Operating Room
- Endoscopy suite
- ESD not being performed
- Other (please specify)

**Q6** After completion of your core gastroenterology/surgical fellowship, how did you train for ESD?

- Fellowship-North America
- Fellowship-overseas
- ASGE or other accredited courses
- Did not pursue further formal ESD training
- Other (please specify)

**Q7** If ESD training was pursued, how long was your cumulative time training?

- <3months
- 3-6months
- 6-12 months
- 12-24months
- >24months
- Not applicable

**Q8** If you performed ESD training, how many ESD procedures did you perform in the following regions?

- Esophagus (enter free text)
- Stomach (enter free text)
- Duodenum (enter free text)
- Colon (enter free text)
- Rectum (enter free text)

**Q9** How many ESD procedures do you currently perform annually in the following regions?

- Esophagus (free text)
- Stomach (free text)
- Duodenum (free text)
- Colon (free text)
- Rectum (free text)

**Q10** Which specialty is performing ESD at your center?

- Gastroenterology
- Surgery
- Both Gastroenterology and Surgery
- Not applicable

**Q11** What is the annual endoscopic mucosal resection (EMR) procedure volume performed by you?

- Upper GI (free text)
- Lower GI (free text)

**Q12** What % of the time are you performing hybrid ESD (using snare for partial resection)?

- I do not perform ESD
- I never use H-ESD
- < 10% of the time
- 10%-50% of the time
- >50% of the time

**Q13** Which of these statements best describes your mindset toward the adoption of ESD? (choose all which apply)

- Ready for adoption by more therapeutic endoscopists
- Need improved training/techniques before significant increase in adoption
- Need more data/guidelines to expand indicated disease states
- Able to increase my volume (pending referrals)
- Reimbursement is constraining adoption
- Cannot increase case volume (capacity constrained)
- Need device improvement before significant increase in adoption
- Other (please specify)

**Q14** Rank the rationale for performing ESD?

- en-bloc resection / definitive diagnosis
- lower recurrence vs. Piecemeal EMR
- Avoiding surgery

**Q15** For which indications are you performing ESD in each of the following anatomies

- Esophagus
  - Low grade dysplasia
  - High grade dysplasia
  - Intramucosal carcinoma
  - Submucosal carcinoma
  - Submucosal tumors
  - Not performing ESD in this anatomy
  - Other (free text)
- Stomach
  - Low grade dysplasia
  - High grade dysplasia
  - Intramucosal carcinoma
  - Submucosal carcinoma
  - Submucosal tumors
  - Not performing ESD in this anatomy
  - Other (free text)
- Duodenum
  - Low grade dysplasia
  - High grade dysplasia
  - Intramucosal carcinoma
  - Submucosal carcinoma
  - Submucosal tumors
  - Not performing ESD in this anatomy
  - Other (free text)
- Colon
  - Low grade dysplasia
  - High grade dysplasia
  - Intramucosal carcinoma
  - Submucosal carcinoma
  - Submucosal tumors
  - Not performing ESD in this anatomy
  - Other (free text)
- Rectum
  - Low grade dysplasia
  - High grade dysplasia
  - Intramucosal carcinoma
  - Submucosal carcinoma
  - Submucosal tumors
  - Not performing ESD in this anatomy
  - Other(free text)

**Q16** What is the recurrence rate from Piecemeal EMR (P-EMR) at your facility?

- **Unknown**
- **No recurrence**
- **<10%**
- **10-25%**
- **25-50%**
- **>50%**

**Q17** In your opinion, what are the greatest challenges of ESD procedures today? (Check all that apply)

- Procedural Time
- Acute perforation risk
- Delayed bleeding
- Delayed perforation risk
- Intraprocedural bleeding
- Device exchanges

**Q18** What are the barriers to adoption for ESD? (please rank)

- Time required for procedure
- Lack of formalized training and/or fellowship training
- Low procedure volume (based on indicated disease state)
- Lack of advanced endoscopic diagnosis knowledge
- Lack of reimbursement
- Lack of society guidelines
- Other (free text)

**Q19** Rank the following for their importance in facilitating ESD adoption to practice in Canada?

- more access to ESD experts
- Better accessories/devices
- ESD courses by CAG
- Creation of an ESD registry
- Other courses (even if not Canadian association of gastroenterology (CAG) based)

**Q20** Where do you see ESD volumes in Canada 5 years from now?

- ESD will no longer be performed
- Decreased
- No significant change compared to now
- Small increase
- Dramatic increase
- Other (free text)

**Q21** What type of injection solution are you typically using for ESD procedures?

- **Saline**
- **Hetastarch (i.e. Voluven, Hespan )**
- **Glycerol**
- **Eleview**
- **Other (please specify-free text)**

**Q22** How satisfied are you with your injection solution?

- Not satisfied
- Somewhat satisfied
- Very satisfied

**Q23** How often are you using tissue retraction techniques?

- Always
- Usually
- Sometimes
- Rarely
- Never

**Q24** Are ESD patients primarily inpatient or outpatient?

- Inpatient
- Outpatient
- Other (please specify-free text)
